# GABAergic integration of transient and persistent neurons in the developing mouse somatosensory cortex

**DOI:** 10.3389/fncel.2025.1556174

**Published:** 2025-02-26

**Authors:** Ahd Abusaada, Federico De Rosa, Heiko J. Luhmann, Werner Kilb, Anne Sinning

**Affiliations:** Institute of Physiology, University Medical Center of the Johannes Gutenberg University, Mainz, Germany

**Keywords:** GABA, synaptogenesis, somatosensory cortex, mouse, development

## Abstract

GABA is an essential element in the function of neocortical circuits. The origin, migration and mechanisms of synaptogenesis of GABAergic neurons have been intensively studied. However, little information is available when GABAergic synapses are formed within the different cortical layers, neuronal cell types and subcellular compartments. To quantify the distribution of GABAergic synapses in the immature somatosensory mouse cortex, GABAergic synapses were identified by spatially coincident immunoprofiles for the pre- and postsynaptic markers vGAT and gephyrin at postnatal days (P)0-12. Between P0-5, GABAergic synapses are mainly restricted to the marginal zone, while at later developmental stages a more homogenous distribution is obtained. Cajal-Retzius neurons represent a major target of GABAergic synapses in the marginal zone with a homogeneous synapse distribution along the dendrite. The number of GABAergic synapses per pyramidal neuron increases substantially between P0 and P12, with a stable density and distribution in basal dendrites. In contrast, along apical dendrites synapses accumulate to more proximal positions after P8. Overall, the results of this study demonstrate that early GABAergic synaptogenesis is characterized by a consistent increase in the density of synapses with first a stringent overrepresentation in the marginal zone and a delayed establishment of perisomatic synapses in pyramidal neurons.

## 1 Introduction

The amino acid γ-amino-butyric-acid (GABA) is a major inhibitory neurotransmitter in the central nervous system and regulates and synchronizes neuronal activity in the mature neocortex (Whittington and Traub, 2003; Mody and Pearce, 2004; Pouille and Scanziani, 2004; Donoso et al., 2018; Barbero-Castillo et al., 2021). In accordance with the variety of GABAergic functions, distinct populations of GABAergic interneurons with specific firing patterns, dendritic morphologies and axonal projection patterns have been identified (Ascoli et al., 2008; Tremblay et al., 2016). Typical examples are parvalbumin-positive (PV) basket cells, which synapse preferentially on the somata and mediate feedforward inhibition, or somatostatin (SST) interneurons, that synapse preferentially on the dendrites and mediate a strong feedback inhibition (Kepecs and Fishell, 2014; Tremblay et al., 2016). Hence, depending on the type of cortical interneurons, GABAergic synapses show distinct spatial distributions, which mediate diverse functions on the network (Favuzzi et al., 2019).

Already during neuronal development, GABA is involved in important functions such as neurogenesis, neuronal migration, differentiation and synaptogenesis (Owens and Kriegstein, 2002; Represa and Ben-Ari, 2005; Cancedda et al., 2007; Luhmann et al., 2014). During these early developmental phases, the immature state of neuronal Cl^–^ homeostasis causes depolarizing membrane responses upon activation of GABA_*A*_ receptors (Yamada et al., 2004; Ben-Ari et al., 2007; Rheims et al., 2008; Blaesse et al., 2009), which can mediate inhibitory but also excitatory actions (Rheims et al., 2008; Kilb, 2021). In accordance with the important developmental role of GABA, the GABAergic system is already functional at perinatal stages. GABAergic neurons are generated at a similar time as pyramidal neurons, migrate from their origin sites in the ganglionic eminences to the neocortex via tangential migration (Marín, 2013; Lim et al., 2018) and settle in the cortical layers already at prenatal stages (Miyoshi and Fishell, 2011). In line with the early presence of GABAergic neurons, the GABA synthetizing enzyme GAD67 can be detected in the perinatal cortex (Behar et al., 1994). Moreover, the presynaptic vesicular GABA transporter (vGAT) (Minelli et al., 2003; Boulland and Chaudhry, 2012) and the postsynaptic GABA receptor anchoring protein gephyrin (Kirsch et al., 1993) are already expressed at perinatal stages, which suggests that GABAergic synapses are formed during these early developmental stages.

More detailed analysis of the functional establishment of GABAergic connectivity was performed in the somatosensory cortex, where the evident topological organization allows to analyze the impact of sensory and/or genetic manipulations on the structural and functional development of neocortical circuits (Kania, 2021; Yang et al., 2018). In the somatosensory cortex, reliable functional GABAergic synapses between SST and pyramidal neurons (Tuncdemir et al., 2016), and fast-spiking PV and pyramidal neurons have been only reported toward the end of the first postnatal week (Daw et al., 2007; see Pangratz-Fuehrer and Hestrin, 2011 and Yang et al., 2014, for a comparable timing in the visual and prefrontal cortex) and a remarkable integration of GABAergic interneurons to cortical networks does not appear before the second postnatal week (Bollmann et al., 2023). In contrast, transient cell populations like Cajal-Retzius neurons (CRn) and subplate neurons (SPn) have functional GABAergic synapses as early as the first postnatal days (Kilb and Luhmann, 2001; Hanganu et al., 2002). Structurally, electron microscopic studies yet revealed clear evidences for symmetric, putative GABAergic synapses around P4, although at rather low densities (Micheva and Beaulieu, 1996; De Felipe et al., 1997; Gour et al., 2021). These studies also showed an increase in the overall density of GABAergic synapses and their number per neuron within the first two postnatal weeks (Micheva and Beaulieu, 1996; Gour et al., 2021; see Virtanen et al., 2018, for a comparable timing in the prefrontal cortex).

In order to provide a detailed view of the spatiotemporal emergence of GABAergic synapses and their spatial organization along the somatodendritic axis of pyramidal neurons during the first postnatal days in the cerebral cortex, we identified GABAergic synapses in the somatosensory cortex of immature mice between P0 and P12. For this purpose, putative GABAergic synapses were identified by colocalization of the pre- and postsynaptic markers vGAT and gephyrin. Individual pyramidal neurons were stained by biocytin filling and CRn by genetic labeling. We found that between P0 and P5 GABAergic synapses are mainly restricted to the marginal zone, with CRn representing a major target of GABAergic synapses in this layer. In deeper layers the density of GABAergic synapses is upregulated between P3 and P8, which is also reflected by the increase in the number of somatic and dendritic synapses on identified pyramidal neurons. The density and distribution of GABAergic synapses on basal dendrites is stable during this developmental period, whereas GABAergic inputs accumulate to more proximal positions on maturing apical dendrites.

## 2 Materials and methods

### 2.1 Slice preparation

All experiments were conducted in accordance with EU directive 86/609/EEC for the use of animals in research and the NIH Guide for the Care and Use of Laboratory Animals, and were approved by the local ethical committee (Landesuntersuchungsanstalt RLP, Koblenz, Germany). All efforts were made to minimize the number of animals and their suffering. Timed-pregnant C57BL/6 mice were obtained from Janvier Labs (Saint Berthevin, France). ΔNp73^*CreIRESGFP*^ (ΔNp73^*Cre*^) (Tissir et al., 2009) and Ai14 line (Strain# 007914) B6.Cg-Gt(ROSA)26Sor^*TM*14(*CAG–tdTomato)Hze*/^J (Madisen et al., 2010) transgenic mice were kept in a C57BL/6J background. All animals were housed in the local animal facility. ΔNp73^*Cre*^ line was crossed with Ai14 reporter line to permanently label CRn. Newborn pups at ages P0 to P12 were deeply anesthetized with enflurane (Ethrane, Abbot Laboratories, Wiesbaden, Germany). After decapitation, the brains were quickly removed and immersed for 2–3 min in ice-cold standard artificial cerebrospinal fluid (ACSF), with the composition (in mM) 125 NaCl, 25 NaHCO_3_, 1.25 NaH_2_PO_5_, 1 MgCl_2_, 2 CaCl_2_, 2.5 KCl, 10 glucose and was equilibrated with 95% O_2_ /5% CO_2_ at least 1 h before use (pH 7.4, osmolarity 306 mOsm). Horizontal slices (400 μm thickness) including the somatosensory cortex were cut on a vibratome (Microm HM 650 V, Thermo Fischer Scientific, Schwerte, Germany). The slices were stored in an incubation chamber filled with oxygenated ACSF at room temperature before they were transferred to the recording chamber or directly fixed for immunohistochemistry.

### 2.2 Whole-cell staining of individual neurons

Individual pyramidal neurons were filled with biocytin via a patch-pipette for subsequent labeling. For this purpose, slices were transferred to a submerged-type recording chamber attached to the fixed stage of a microscope (BX51 WI, Olympus). Pyramidal neurons in the somatosensory cortex were identified by their location and appearance in infrared differential interference contrast image. Whole-cell patch-clamp recordings were performed as described previously (Achilles et al., 2007) at 31 ± 1°C. Patch-pipettes (5–12 MΩ) were pulled from borosilicate glass capillaries (2.0 mm outside, 1.16 mm inside diameter, Science Products, Hofheim, Germany) on a vertical puller (PP-830, Narishige). Patch-pipettes were filled with a solution containing (in mM) 128 K-gluconate, 2 KCl, 4 NaCl, 1 CaCl_2_, 11 EGTA, 10 K-HEPES, 2 Mg_2_-ATP, 0.5 Na-GTP and 2 lidocaine-N-ethyl chloride (pH adjusted to 7.4 with KOH and osmolarity to 306 mOsm with sucrose). On the day of experiment ∼0.5% biocytin (Sigma, Taufkirchen, Germany) was added to the pipette solutions for labeling of the recorded neurons.

Signals were recorded with a discontinuous voltage-clamp/current-clamp amplifier (SEC05L, NPI, Tamm, Germany), low-pass filtered at 3 kHz and stored and analyzed using an ITC-1600 AD/DA board (HEKA) and TIDA software. All voltages were corrected post-hoc for liquid junction potentials of −9 mV. Cells were kept under whole-cell conditions for at least 15 min, to allow proper staining of dendritic and axonal compartments. Up to 2 cells per slice were filled with biocytin. Sliced were subsequently transferred in 4% paraformaldehyde solution (ROTI^®^ Histofix 4%, Carl Roth) and fixed for 2–3 h.

### 2.3 Immunohistochemistry

After fixation slices were washed in phosphate buffer and incubated in blocking solution [7% normal donkey serum, 0.8% Triton, 0.05% sodium azide in phosphate buffered saline (PBS)] for 2 h at room temperature or overnight at 4°C. Slices were incubated for about 65 h at 4°C in PBS (containing 0.05 sodium Azide /0.3% Triton) with the following primary antibodies: rabbit monoclonal anti-Gephrin (147018, Synaptic Systems, 1:500) and guinea pig polyclonal anti-vGAT (131004, Synaptic Systems, 1:500). Subsequently, DAPI and the following fluorophore-conjugated secondary antibodies were used: Cy3- or Alexa Fluor 488- conjugated AffiniPure F(ab)2 donkey anti-rabbit IgG (711-166-152, Jackson ImmunoResearch 1:200 or 711-546-152, Jackson ImmunoResearch 1:200), Alexa Fluor 647-conjugated AffiniPure F(ab)2 donkey anti-guinea pig (706-606-148, Jackson ImmunoResearch 1:200). In case of biocytin-filled cells, slices were incubated with Alexa Fluor 488-conjugated streptavidin (S11223, Molecular Probes, ThermoFisher Scientific). Slices were embedded in Fluoromount-G (SBA-0100-01, Biozol).

### 2.4 Imaging

Immunofluorescence was investigated with a spinning disk confocal system (5 Elements, Visitron, Puchheim, Germany), consisting of a Yokogawa spinning disk head (CSU-W1, 50 μm pinholes) attached to a Nikon Ti2-E microscope body equipped with a 60× water immersion objective (CFI Plan Apo VC 60XWI, NA 1.20, Nikon) and a Prime BSI sCMOS camera (2048 × 2048 pixels, 6.5 μm pixel size, Photometrics) controlled by VisiView software (Visitron). Excitation of fluorescence was conducted at 405 nm, 488 nm, 561 nm and 640 nm utilizing diode lasers emitting at 405 nm/488 nm/640 nm (Toptica) and a diode pumped laser (DPL) at 561 nm (Cobolt). The emitted fluorescence was filtered by bandpass filters 460/50 (Chroma ET460/50) for 405 nm excitation, 525/50 (Chroma ET525/50) for 488 nm excitation, 623/32 (Semrock FF01-623/32) for 561 nm excitation, and a 633 longpass filter (Semrock BLP01-633R) for 640 nm excitation. The plane and stack parameters were adjusted depending on the depth and length of the dendrite, maintaining a consistent z-distance between planes of 0.5 μm and a 10% overlap between stacks.

### 2.5 Image analysis

Fluorescence images were resized by reducing the resolution from 16 to 8 bit and 2×2 binning. Subsequently, the images were stitched, using the Grid Collection Stitching function implemented in ImageJ (Preibisch et al., 2009). Determination of the density of putative synaptic sites was performed in ImageJ. For this purpose, a thorough background correction using a rolling ball algorithm with a size of 50 pixel was performed, followed by a binarization using a visually determined threshold (in most images amounting to mean + 1.5 to 3 × SD). Particles with a size of 5 to 50 pixel^2^ (corresponding to 0.24–2.42 μm^2^) were considered as relevant profiles. Subsequently, putative synapses were assigned when profiles of postsynaptic gephyrin sites and presynaptic vGAT sites either overlap or showed a distance of maximal 1 pixel (corresponding to a distance of 0.33 μm).

Identification of synapses aligned to the surface of neurons was performed using the IMARIS 9.9.1 software (Oxford instruments). The layer-wise CRn reconstruction was obtained with the Surface function of IMARIS. As for the single cell reconstruction, the dendritic compartment of both pyramidal cells and CRn was made with the Filament Tracer function, while the somata were made with the Surface function. Presynaptic (vGAT positive) and postsynaptic (gephyrin positive) profiles were modeled using the Spot function with spots radii of 0.5 μm. For the identification of putative GABAergic synapses, postsynaptic profiles were filtered to fall within an absolute distance of 0.33 μm to vGAT positive spots. Putative synapses located within an absolute distance of maximum 1 μm to the dendrite or soma surface were assigned to the cell. Metrics of reconstructed cells with dendritic compartments and synapse localizations were defined and calculated as follows (see also Supplementary Figure 1): total dendritic length was calculated as the sum of dendritic lengths, dendritic order was defined as branching point level and maximal dendritic order reflected the highest dendritic order per cell. Cortical depth was described as the absolute distance of pyramidal neuron soma to the pial surface. Synapse density was calculated by dividing the total number of dendritic synapses by the total dendritic length. The relative distance to the soma was calculated by dividing the distance of a synapse to the soma by the maximal synapse distance to the soma found per cell, with synaptic distance defined as the distance between a synapse and the soma along the dendritic path.

### 2.6 Statistics

Values are expressed as mean values ± SEM. For larger data sets (with *n* > 20) median with 25% and 75% percentile are displayed for clarity. All statistical tests were performed using GraphPad Prism 10 (GraphPad). Normality of sample distributions was tested with Shapiro–Wilk test and homogeneity of variances was tested with Bartlett’s test. Comparisons between multiple groups were made either with a one-way ANOVA followed by Tukey’s multiple comparison post-hoc test for data with a normal distribution, or a Kruskal–Wallis test followed by Dunn’s multiple comparison post-hoc test, if data did not pass the normality and/or homogeneity of variances test. Comparisons between two groups were made with Student’s unpaired *t*-test. Significance was considered at *p*-values < 0.05.

## 3 Results

**FIGURE 1:**
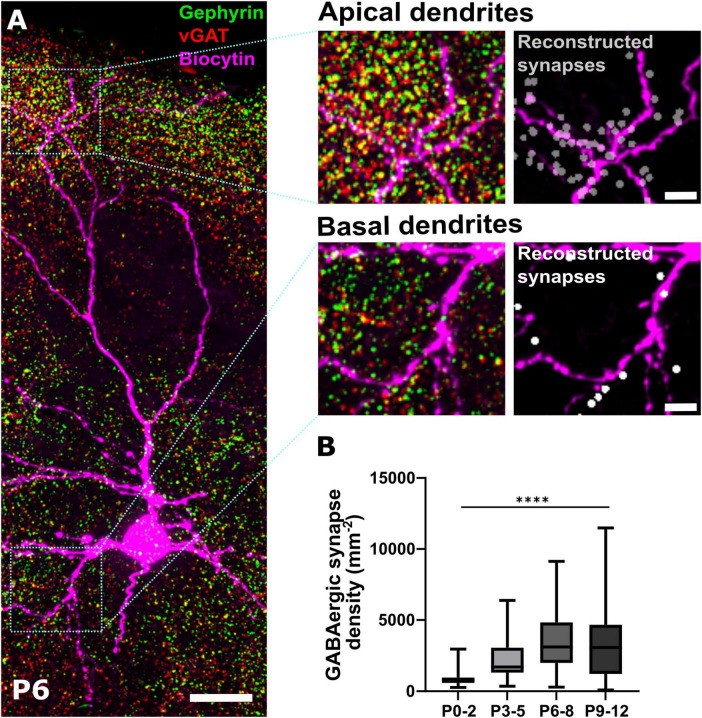
Immunohistochemical identification of GABAergic synapses. **(A)** Representative immunohistochemical staining of somatosensory cortex against gephyrin and vGAT at P6. Colocalization analysis in combination with biocytin filling of pyramidal neurons allows the identification of GABAergic synapses along apical and basal dendritic processes shown as reconstructed synapses on the right. Overview, scale bar = 20 μm, insets, scale bar = 5 μm. **(B)** Quantification of overall density of GABAergic synapses across ROIs covering the entire depth of the somatosensory cortex during early postnatal development. Note, that the density of GABAergic synapses significantly increases from P0-2 until P6-8. ****p < 0.0001 (Kruskal–Wallis test).

In order to quantify the spatial distribution of GABAergic synapses during the first 12 postnatal days, we performed immunohistochemical stainings against the presynaptic marker vGAT and the postsynaptic marker gephyrin. Based on high resolution confocal images spanning all laminae of the somatosensory cortex, putative GABAergic synapses were identified as spatially coincident immunopositive profiles (Figure 1A). For further analysis of the distribution of GABAergic synapses along the somatodendritic axis of neurons, pyramidal cells were optically-identified and labeled by intracellular injection with biocytin via the patch-clamp pipette (Figure 1A) or, in a subset of experiments, CRn were labeled via cell-type specific overexpression of a reporter gene.

### 3.1 Properties of the recorded cells

**FIGURE 2:**
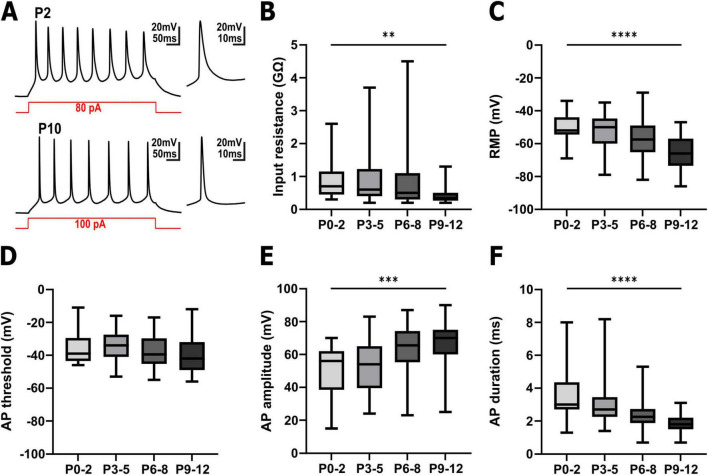
Electrophysiological properties of pyramidal neurons in the immature somatosensory cortex between P0 and P12. **(A)** Representative voltage traces recorded in pyramidal neurons from a P2 (upper trace) and a P10 (lower trace) cortical slice. The snippets on the right depict the first action potential (AP) at a higher temporal resolution. **(B)** The input resistance significantly decreased with postnatal age. **(C)** The resting membrane potential (RMP) was significantly shifted to hyperpolarizing direction with postnatal age. **(D)** The AP threshold was not significantly altered during the investigated interval. **(E)** The AP amplitude significantly increased with postnatal age. **(F)** The AP duration significantly shortened with postnatal age. **p < 0.01, ***p < 0.001, ****p < 0.0001 (Kruskal–Wallis test or one-way ANOVA).

In total, 117 pyramidal cells were successfully patched for this study (Figure 2 and Supplementary Table 1). These neurons displayed the typical properties of immature neocortical pyramidal neurons (Figure 2A). At P0-2 the patched neurons had an input resistance of 0.9 ± 0.13 GΩ (*n* = 25 cells), which significantly (Figure 2B, Kruskal–Wallis test, *p* = 0.0023) decreased to 0.45 ± 0.07 GΩ (*n* = 24 cells) at P9-12. The resting membrane potential (RMP) of the cells significantly (Figure 2C, one-way ANOVA, *p* < 0.0001, F = 8.777) hyperpolarized from −50.7 ± 1.8 mV (*n* = 25 cells) at P0-2 to −65.4 ± 2.0 mV (*n* = 24 cells) at P9-12. The majority of the patched neurons were able to generate action potentials (AP) upon injection of a depolarizing current (Figures 2A, D–F). While at P0-2 the APs showed rather immature properties with an amplitude of 49.9 ± 3.5 mV and a halfwidth of 3.6 ± 0.36 ms (*n* = 21 cells), a maturation of these AP properties occurred until P9-12 (66.2 ± 3.2 mV amplitude and 1.9 ± 0.12 ms halfwidth, *n* = 23 cells). The AP threshold was not significantly altered during the observed interval (Figure 2D). In summary, the passive and active properties of the recoded pyramidal neurons are in line with their continuous functional maturation (LoTurco et al., 1995; Luhmann et al., 2000).

### 3.2 Spatial profile of GABAergic synapses during the first postnatal days

**FIGURE 3:**
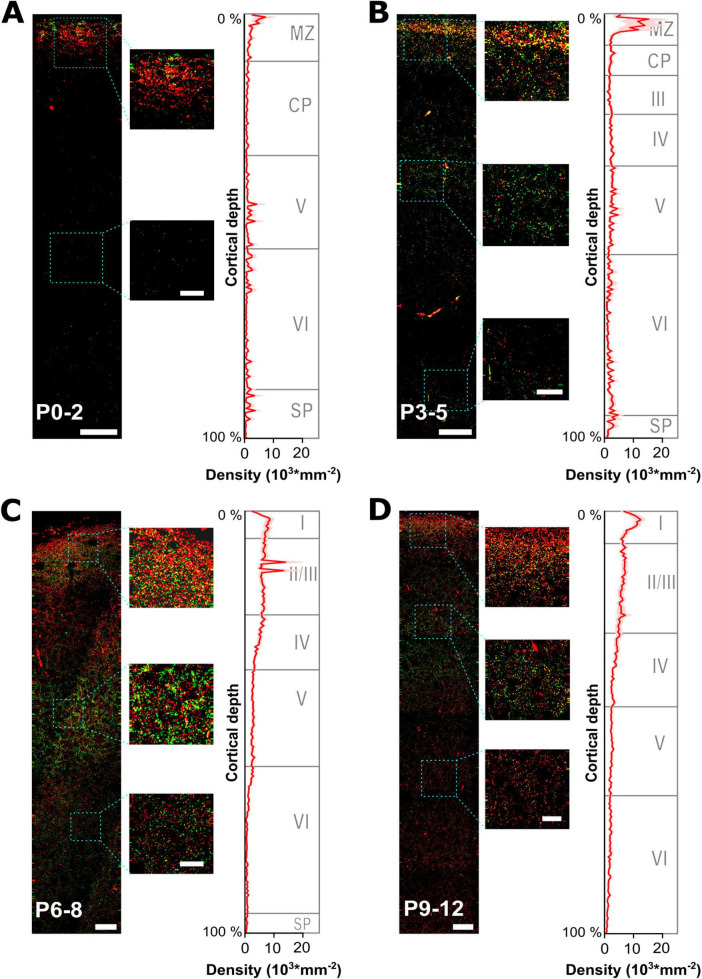
Developmental profiling of GABAergic synapses maturation across all layers of the somatosensory cortex. **(A–D)** Representative gephyrin (green) and vGAT (red) immunohistochemical stainings at 4 different developmental time points from P0 to P12. The small insets illustrate characteristic fluorescence signals at indicated regions. Respective graphs on the right display the estimated density of GABAergic synapses (identified by gephyrin and vGAT colocalization) plotted versus the relative cortical thickness (calculated from pial surface to the beginning of the subventricular zone/white matter) per age group. The approximate boundaries of cortical layers in the somatosensory cortex were indicated as published for P0, P4 and P6 (Ignacio et al., 1995) and at P10 (Imura et al., 2005). MZ, marginal zone; CP, cortical plate; SP, subplate. Note the high density of GABAergic synapses in the MZ at P0-5, while during later developmental stages GABAergic synapses become prominent also at lower cortical layers. Overview, scale bar = 25 μm, insets, scale bar = 10 μm.

In total, 66 slices were used for immunohistochemical labeling of GABAergic synapses. In each slice up to four non-overlapping regions of interest (ROI), spanning perpendicularly from the pial surface to the lower cortical border, were defined and analyzed (see Figure 3). Through the identification of spatially coincident vGAT and gephyrin profiles, we detected 2163 putative GABAergic synapses (*n* = 24 ROIs in 9 slices) at P0-2, 18764 putative GABAergic synapses (*n* = 49 ROIs in 13 slices) at P3-5, 64661 putative GABAergic synapses (*n* = 105 ROIs in 24 slices) at P6-8, and 64456 putative GABAergic synapses (*n* = 71 ROIs in 20 slices) at P9-12.

The quantification of GABAergic synapse density across the entire ROIs, representing the average density of GABAergic synapses within the somatosensory cortex, revealed a significant (Kruskal–Wallis test, *p* < 0.0001) upregulation during the first postnatal days (Figure 1B). The calculated density of GABAergic synapses increased with postnatal development from 905.7 ± 126.2 mm^–2^ (*n* = 24 ROIs) at P0-2, to 2,402.1 ± 239.7 mm^–2^ (*n* = 49 ROIs) at P3-5, and to 3,555.5 ± 212.4 mm^–2^ (*n* = 105 ROIs) at P6-8. The synapse density at P9-12 amounted to 3,531.0 ± 315.0 mm^–2^ (*n* = 71 ROIs) and was not significantly (*p* = 0.99, Dunn’s Post-hoc test) different from P6-8.

To analyze the spatial profile of GABAergic synapses, we next quantified the density profile of GABAergic synapses in these ROIs along the relative cortical depths, i.e., 200 perpendicular bins spanning the total distance between pial surface and white matter (Figure 3). These analyses revealed that at P0-2 the highest density (4,723 ± 776 mm^–2^, *n* = 24 ROIs) was observed in the upper layer, representing the marginal zone (Figure 3A). Already 17.9% of all GABAergic synapses were located in the uppermost 5% of the cortex. The density of GABAergic synapses was lower (654 ± 45 mm^–2^) between 10 and 40% of the cortical depth, reflecting mainly the cortical plate and part of layer V during these developmental stage (Ignacio et al., 1995). In lower layers, the density of synapses was slightly higher with a local maximum in the lowest 10% of the cortex (on average 704 ± 228 mm^–2^). In P3-5 slices, the spatial profile of GABAergic synapses remained comparable but the overall density increased (Figure 3B). The highest density of GABAergic synapses was still observed in the most external cortical layer (9,799 ± 2,138 mm^–2^, *n* = 49 ROIs), with 17.8% of all synapses located in the uppermost 5% of the cortex. Moreover, a considerable increase in the density of GABAergic synapses was observed for the cortical layers below (2,209 ± 62 mm^–2^ for 10–40% and 1,840 ± 326 mm^–2^ for 40.5–89.5% of cortical thickness). The average synapse density in the lowest 10% of the cortex also increased to 2,351 ± 9,465 mm^–2^.

At P6-8, GABAergic synapses showed a considerably altered spatial profile (Figure 3C). Although a high density of GABAergic synapses was observed close to the pial surface (6,004 ± 1,150 mm^–2^, *n* = 105 ROIs, i.e., 10.6% of all GABAergic synapses were located in the uppermost 5% of the cortex), the synapse density in the cortical layers below substantially increased. In putative supragranular layers (10–25% of cortical depth) the average density of GABAergic synapses was 6,722 ± 350 mm^–2^. Whereas, the density decreased in lower cortical layers (50–90% of cortical depth, reflecting infragranular layers) to 1,517 ± 100 mm^–2^, with the lowest level observed close to the white matter (805 ± 46 mm^–2^ in the lowest 10% of cortical thickness). A comparable distribution was also observed in P9-12 slices (Figure 3D), with a density of 10,000 ± 1,028 mm^–2^ (*n* = 71 ROIs) close to the pial surface, a density of 6,251 ± 118 mm^–2^ in putative supragranular layers (10–25% of cortical depth), 2,007 ± 45 mm^–2^ in putative infragranular layers (50–90% of cortical depth), and 1,021 ± 63 mm^–2^ in the lowest 10% of cortical thickness.

In summary, these analyses revealed a substantial alteration in the distribution of GABAergic synapses, with a prominent restriction of GABAergic synapses to the uppermost cortical zone between P0-5. At later developmental stages a more homogenous distribution of GABAergic synapses developed across the cortical depth, even though a higher density of GABAergic synapses in upper cortical layers was retained.

### 3.3 Cajal-Retzius neurons are a major target of GABAergic synapses in the marginal zone

The prior results showed a high prevalence of GABAergic synapses during the first 5 postnatal days in the uppermost cortical layer, which corresponds to the marginal zone (Ignacio et al., 1995; Bystron et al., 2008). Since CRn represent a major neuronal cell type in the marginal zone (Kirischuk et al., 2014; Causeret et al., 2021), and receive mainly GABAergic synaptic inputs (Kilb and Luhmann, 2001; Radnikow et al., 2002; Kirmse et al., 2006; Damilou et al., 2024), we hypothesized that a considerable fraction of theses GABAergic synapses are attached to CRn.

**FIGURE 4:**
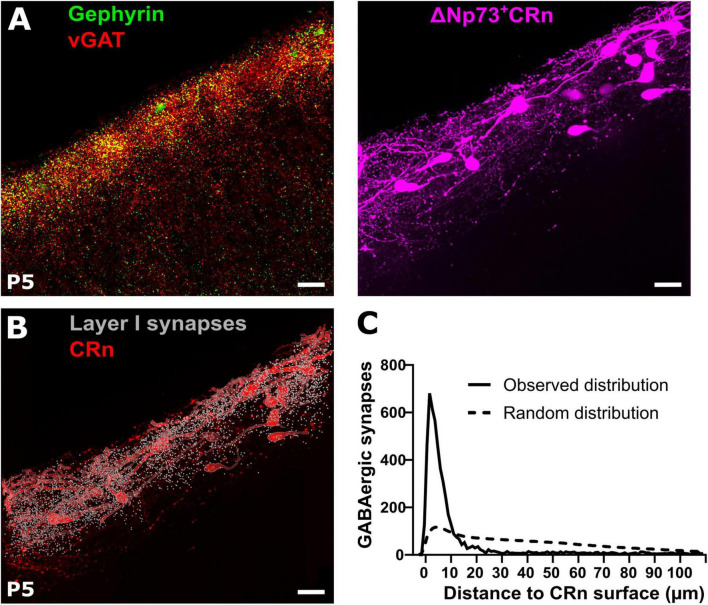
Reconstruction of GABAergic synapses and Cajal-Retzius neurons (CRn) in the marginal zone. **(A)** Representative gephyrin and vGAT co-staining (left) and tdTomato reporter signal in ΔNp73^Cre^ CRn (right) at P5. **(B,C)** Corresponding reconstruction of ΔNp73-positive CRn with layer 1 GABAergic synapses and quantification of the relative distances between identified GABAergic synapses and CRn on the right. Note, that the majority of GABAergic synapses is in close proximity to CRn surfaces, which is much higher than predicted by the Complete Spatial Random (CSR) distribution of synapse locations (dashed line). Scale bar = 20 μm.

In order to quantify GABAergic synapses on CRn we next identified vGAT and gephyrin double-positive profiles in slices from P5 ΔNp73^Cre+^ mice (Tissir et al., 2009) crossbred with reporter Ai14 mice. In these mice the majority of CRn, representing the hem- and septum-derived CRn (Bielle et al., 2005; Causeret et al., 2021), can be identified by a tdTomato reporter signal (Figure 4A). To enable the identification of putative GABAergic synapses on CRn, we determined the surface of tdTomato^+^ cells as well as the profiles of putative GABAergic synapses and then calculated the distances between each putative GABAergic synapse in the marginal zone to its closest surface of a tdTomato^+^ cell (Figure 4B). The distance distribution analyses revealed that the putative GABAergic synapses were closer to CRn surface than expected, if a random distribution of synaptic spots was assumed (Figure 4C), suggesting a substantial synapse formation onto CRn. This finding was supported by the observations that > 30% of all synaptic spots were located at a distance of less than 2.1 ± 0.4 μm (calculated from the center of the synaptic spots and thus including their radius of 0.5 μm) from the nearest surface of a CRn, meaning that > 30% of all synaptic spots were located at an absolute distance of less than 1.6 ± 0.4 μm (Supplementary Figure 2A, B). If we assessed synaptic spots with a distance of ≤ 1.0 μm to the tdTomato surface as GABAergic synapses onto CRn, 21.8 ± 3.6% of putative GABAergic synapses in layer 1 (7,940 ± 2,210 synaptic spots in *n* = 8 slices) are attached to CRn (Supplementary Figure 2C).

Taken together, this data suggests that CRn indeed represent a major synaptic target of GABAergic afferents to the marginal zone at early postnatal development.

### 3.4 Localization of GABAergic synapses on single pyramidal neurons

Next, we analyzed whether the sparse synaptic spots observed in the cortex below the marginal zone represent early GABAergic synaptic contacts to pyramidal cells and how the cellular localization of these contacts change with development. In this respect, we reconstructed the surface of the somatodendritic compartments of individual pyramidal cells identified by biocytin-based labeling (Figure 1A). From the 117 biocytin-filled neurons a sufficient reconstruction of basal and apical dendrites was only possible for 32 neurons.

**FIGURE 5:**
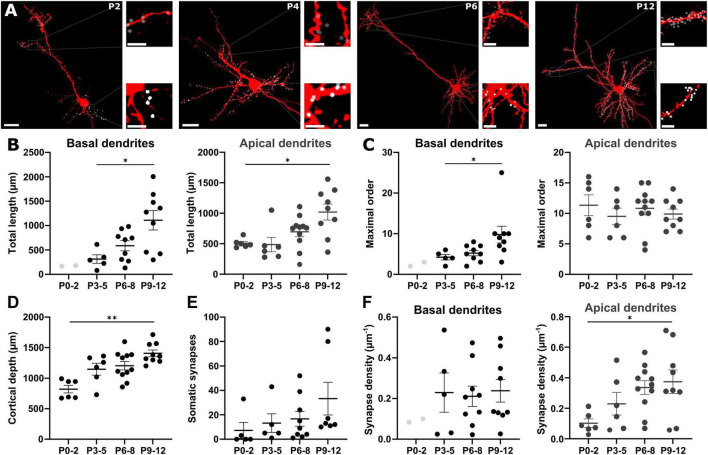
Localization of GABAergic synapses on pyramidal neurons. **(A)** Representative biocytin-filled pyramidal neurons at different postnatal stages with reconstructed GABAergic synapses on the apical (gray dots) and the basal (white dots) dendrites. Overview, scale bar = 20 μm, insets, scale bar = 5 μm. **(B)** The total length of both apical and basal dendrites significantly increases with postnatal age. **(C)** Only for basal dendrites ramification complexity significantly increases during early development. **(D)** Cortical depth increases during early development. **(E)** The number of somatic GABAergic synapses onto pyramidal neurons significantly increases from P0 to P12. **(F)** During early development, GABAergic synapse density along basal dendrites does not change while the density at apical dendrites significantly increases with postnatal age. Data points for P0-2 basal dendrites are displayed in B, C and F but not included in analysis. *p < 0.05; **p < 0.01 (Kruskal–Wallis or one-way ANOVA).

During the first 12 postnatal days, apical dendrites underwent a significant (Kruskal–Wallis test, *p* = 0.0102) elongation from 508 ± 30 μm at P0-2 (*n* = 6 cells) to 1,020 ± 130 μm at P9-12 (*n* = 9 cells, Figures 5A, B and Supplementary Table 2), while the complexity of the dendritic tree, as quantified by the average maximal order of dendritic branches, remained constant (Figure 5C). Conversely, in basal dendrites the development of a complex dendritic structure was more prominent. However, a complete reconstruction of basal dendrites at P0-2 was only possible for 2 neurons, which were thus not included in statistical analysis. From P3-5 on, a significant (Kruskal–Wallis test, *p* = 0.0144) increase in the total length from 313 ± 88 μm at P3-5 (*n* = 5 cells with basal dendrites) to 1,109 ± 200 μm at P9-12 (*n* = 9 cells, Figure 5B) was evident. The average maximal order significantly (Kruskal–Wallis test, *p* = 0.0127) increased from 4.2 ± 0.7 at P3-5 to 9.8 ± 2.1 at P9-12 (Figure 5C). As expected, cortical depth constantly increased during early postnatal development in a significant way (Kruskal–Wallis test, *p* = 0.0022) from 822 ± 63 μm at P0-2 (*n* = 6) until 1,409 ± 55 μm at P9-12 (*n* = 9, Figure 5D).

The number of somatic GABAergic synapses was 7 ± 6 (*n* = 5 cells) at P0-2 compared to 13 ± 8 (*n* = 5 cells) at P3-5, 17 ± 6 (*n* = 9 cells) at P6-8, and 33 ± 13 (*n* = 7 cells) at P9-12 (Figure 5E). The average number of synapses along apical dendrite increased from 52 ± 14 at P0-2 (*n* = 6) to 94 ± 25 at P3-5 (*n* = 6), 240 ± 46 at P6-8 (*n* = 11), and 427 ± 99 at P9-12 (*n* = 9). In basal dendrites, the number of synapses increased from 84 ± 33 at P3-5 (*n* = 5) to 100 ± 30 at P6-8 (*n* = 9), and 294 ± 92 at P9-12 (*n* = 9). However, this massive increase in the number of GABAergic synapses in the basal dendrites was balanced by the dendritic elongation during this period. Thus, the average density of GABAergic synapses at basal dendrites was maintained at stable levels (0.2 ± 0.1 μm^–1^ at P3-5, 0.2 ± 0.05 μm^–1^ at P6-8, and 0.2 ± 0.06 μm^–1^ at P9-12, one-way ANOVA *p* = 0.9448, F = 0.0570, Figure 5F). In contrast, a significant (one-way ANOVA, *p* = 0.0275, F = 3.533) increase of the GABAergic synapse density from 0.1 ± 0.03 μm^–1^ at P0-2, to 0.2 ± 0.07 μm^–1^ at P3-5, 0.3 ± 0.04 μm^–1^ at P6-8, and 0.4 ± 0.08 μm^–1^ at P9-12 was observed in apical dendrites (Figure 5F).

**FIGURE 6:**
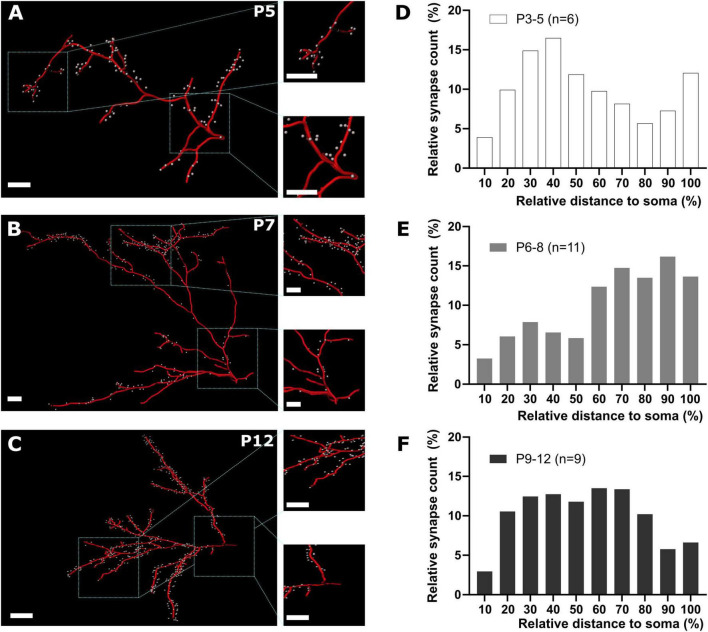
GABAergic synapse distribution on apical dendrites during early cortical development. **(A–C)** Representative reconstructions of GABAergic synapse positions on apical dendrites at different postnatal time points. Overview, scale bar = 10 μm, insets, scale bar = 10 μm. **(D–F)** Quantification of GABAergic synapse distribution along apical dendrites between P3 and P12 plotted as relative number of synapses against relative distance to the soma. Note that from P3-5 to P6-8 the relative prevalence of GABAergic synapses shifts from the soma to distal locations **(D,E)**, while at P9-12 GABAergic synapses are equally distributed along the apical dendrites **(F)**.

**FIGURE 7:**
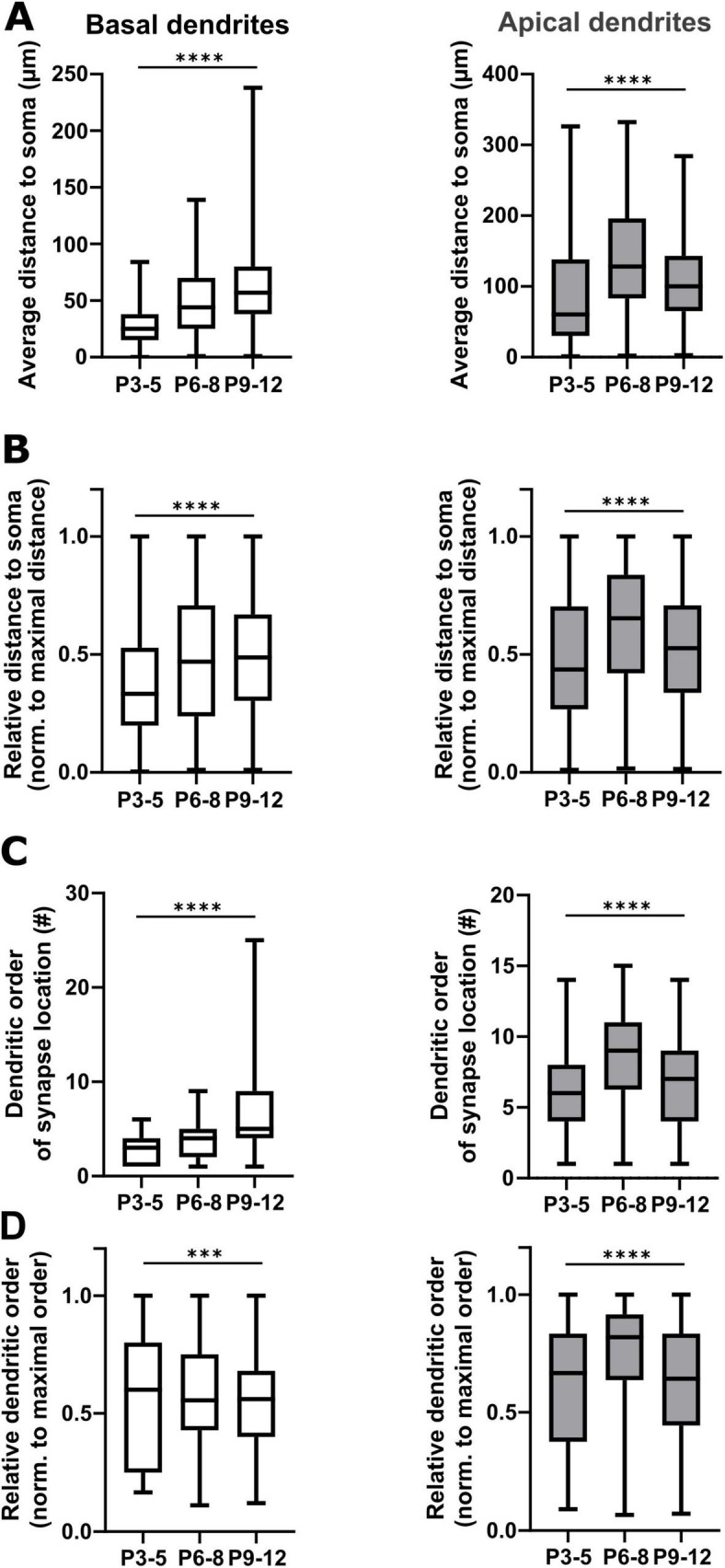
Comparative analyses of GABAergic synapse positions on basal and apical dendrites. **(A)** Quantification of absolute position of GABAergic synapses along basal and apical dendrites. Note that along basal dendrites the median distance of the GABAergic synapses to the soma of pyramidal neurons constantly increases until P12, while in the apical dendrites it declines after P8. **(B)** The relative distance (normalized to the maximal synapse distance) of GABAergic synapses to the soma along basal and apical dendrites follows the same developmental trajectory. **(C)** Quantitative analyses of dendritic order of GABAergic synapse positions revealed that median dendritic order of synapses located at basal dendrites increased with postnatal age, while apical dendritic order decreased after P6-8. **(D)** Accordingly, the median relative dendritic order of synapses located on basal dendrites (normalized to maximal order per cell) is mostly stable with a slight increase from P6-8 until P9-12, while the order of GABAergic synapse locations on apical dendrites first increases until P6-8 and then significantly drops. ***p < 0.001; ****p < 0.0001 (Kruskal–Wallis Test).

Next, we analyzed whether the relative position of GABAergic synapses along the dendrites of pyramidal neurons changes with postnatal development. Due to the low density of GABAergic synapses found in the first two postnatal days, we further only included P3-12 slices in these analyses. Plotting the fraction of GABAergic synapses along basal dendrites against the relative distance to the soma demonstrated that the relative distance from the soma increased from the first 10 to 60% at P3-5 to an almost even distribution between 20 to 90% at P6-8. Later on, at P9-12 most synapses were localized at a relative distance of 30 to 60% (Supplementary Figure 3). In apical dendrites at P3-5 a high fraction of synapses was also located between the first 20 to 50% of the dendritic path (Figures 6A, D). In contrast, at P6-8 the majority of the synapses was located in the most distal parts of the apical dendrite (≥ 60%, Figures 6B, E) and at P9-12 GABAergic synapses were more evenly distributed along the apical dendrite (Figures 6C, F). This biphasic change in the distribution of GABAergic synapses on apical dendrites was also obvious in the quantitative analysis, where the average distance of GABAergic synapses to the soma showed a significant (Kruskal–Wallis test, *p* < 0.0001) alteration during the postnatal development, increasing from 96 ± 4 μm at P3-5 to 143 ± 2 μm at P6-8, but decreasing again to 111 ± 1 μm at P9-12 (Figure 7A). In the basal dendrites, the average distance of GABAergic synapses to the soma consistently (Kruskal–Wallis test, *p* < 0.0001) increased from 29 ± 1 μm at P3-5 to 70 ± 1 μm at P9-12 (Figure 7A). Even when we considered the prominent elongation of basal dendrites by normalizing the synaptic distance to the maximum distance detected in each neuron, a significant (Kruskal–Wallis test, *p* < 0.0001) shift to more distal locations of GABAergic synapses from P3-5 to P6-8 remained (Figure 7B). Conversely, in the apical dendrites the normalized synaptic distance displayed again a significant (Kruskal–Wallis test, *p* < 0.0001) biphasic change (Figure 7B). The differential profile of basal and apical dendrite development was also evident when dendritic order of GABAergic synapse locations was compared across age groups. GABAergic synapses were located at significantly (Kruskal–Wallis test, *p* < 0.0001) higher order from P3-5 (3.0 ± 0.08) to P9-12 (7.0 ± 0.09) on basal dendrites, while on the apical dendrites the order significantly (Kruskal–Wallis test, *p* < 0.0001) increased from 6.4 ± 0.12 at P3-5 to 9.0 ± 0.07 at P6-8 and then decreased to 7.1 ± 0.05 at P9-12 (Figure 7C). Accordingly, when the dendritic order of GABAergic synapses was normalized to the maximum one detected per cell, apical dendrites displayed a significant (Kruskal–Wallis test, *p* < 0.0001) increase from simpler (0.63 ± 0.01 at P3-5) to more complex dendritic compartments (0.76 ± 0.004) at P6-8 before decreasing at P9-12 (0.64 ± 0.004). In contrast, on basal dendrites (Figure 7D) the relative order of GABAergic synapses was mostly stable with only a minor but significant (Kruskal–Wallis test, *p* = 0.0003) decline from P3-5 (0.57 ± 0.01) until P9-12 (0.55 ± 0.004).

In summary, these observations revealed a consistent increase in the number of GABAergic synapses on pyramidal neurons between P0 and P12. In basal dendrites the increase in the number of synapses was proportional to the increase in total dendritic length, thus resulting in a stable density and distribution of GABAergic synapses. Conversely in apical dendrites, the density of synapses and their localization followed a more complex trajectory, resulting in an accumulation of GABAergic synapses to more proximal parts locations P12.

### 3.5 Localization of GABAergic synapses at single Cajal-Retzius neurons

The earlier results suggest a considerable GABAergic input to CRn, in line with previous GABAergic synaptic currents in electrophysiological recordings. On the other hand, the reported frequency and amplitude of GABAergic inputs to CRn is relatively low (Kilb and Luhmann, 2001; Radnikow et al., 2002; Kirmse et al., 2006). Thus, we next investigated the absolute number of putative GABAergic synapses onto individual CRn. For this analysis we analyzed 11 CRn (from *n* = 8 slices), for which a mostly complete somatodendritic reconstruction could be revealed.

**FIGURE 8:**
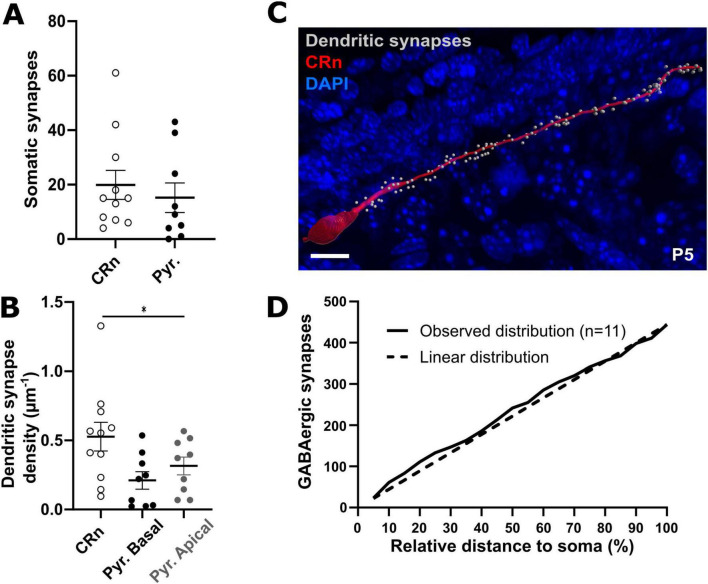
Comparison of GABAergic synapse density and dendritic distribution between pyramidal neurons and CRn at P4-6. **(A)** Quantification of somatic GABAergic synapses shows similar numbers of GABAergic synapses between CRn and pyramidal neurons at P4-6. **(B)** CRn dendrites show a higher density of synapses compared to apical and basal dendrites of pyramidal neurons. **(C)** Representative reconstruction of a P5 CRn and its GABAergic dendritic synapses. Scale bar = 5 μm **(D)** GABAergic synapses are linearly distributed along the dendrites of CRn. *p < 0.05 (one-way ANOVA).

The average number of putative GABAergic synapses on the soma of CRn at P4-6, defined as synaptic spots within a distance of ≤ 1.0 μm to the surface, was 20 ± 5, and thus comparable to the number of somatic synapses in pyramidal neurons at the same age (Figure 8A). The average length of the reconstructed dendrite in these cells was 70.7 ± 6.4 μm. On average, 40 ± 13 synaptic spots were located at a distance of ≤ 1.0 μm to the dendritic surface, resulting in a density of putative GABAergic synapses of 0.53 ± 0.1 μm^–1^ (Figure 8B). This density is significantly higher (one-way ANOVA, *p* = 0.0343, F = 3.849) as compared to the density observed in basal and apical dendrites of P4-6 pyramidal neurons. The distribution of GABAergic synapses along the dendrite was surprisingly homogeneous (Figure 8C), and nearly followed a linear distribution along the dendritic length (Figure 8D).

In summary, these experiments revealed a relatively high density of GABAergic synapses on CRn with a homogeneous distribution along their dendrites.

## 4 Discussion

The aim of the present study was to investigate the development of GABAergic synapses in the early postnatal somatosensory cortex, both across cortical layers and along the somatodendritic axis of individual neurons. The main findings of this study can be summarized as follows: (i) GABAergic synapses are mainly restricted to the marginal zone between P0-5, while during later developmental stages a more homogenous distribution is obtained. (ii) CRn represent a major target of GABAergic synapses in the marginal zone. (iii) The density of GABAergic synapses onto CRn is relatively high with a homogeneous distribution along their dendrites. (iv) The number of GABAergic synapses per pyramidal neuron increases substantially between P0 and P12, with a mostly stable density and distribution in basal dendrites. In contrast, GABAergic synapse density increases in apical dendrites with a biphasic trajectory and a condensation to more proximal locations after P8.

The identification of GABAergic synapses in this study is based on a close spatial correlation between profiles for the presynaptic marker vGAT and the postsynaptic marker gephyrin. The spatial dimensions used for the identification of clusters (approximately 0.2–2 μm^2^) are in line with the described minimal size of gephyrin clusters (Arama et al., 2015; Virtanen et al., 2018) and the size range of presynaptic terminals during development (Karube et al., 2004; Arama et al., 2015). The transmembrane protein vGAT, also known as vesicular inhibitory amino acid transporter (VIAAT), is expressed in synaptic vesicles and uses the proton gradient to accumulate the inhibitory neurotransmitter GABA, as well as glycine (Gasnier, 2004). The cytoplasmic protein gephyrin is an important element of the postsynaptic complex that anchors, beside GABA_A_ receptors, glycine receptors in the postsynaptic membrane (Kuhse et al., 1995; Pizzarelli et al., 2020). Hence, the colocalization of both proteins could theoretically indicate GABAergic and glycinergic synapses. While in the mature nervous system glycinergic synapses are nearly exclusively found in the brainstem and the spinal cord (Kuhse et al., 1995), functional glycine receptors have been described in the early postnatal cerebral cortex (Malosio et al., 1991; Flint et al., 1998; Okabe et al., 2004). Therefore, we cannot exclude that a small fraction of the synapses identified in our study also represents glycinergic synapses. However, glycine receptors in the immature neocortex are mainly activated by non-synaptic mechanisms (Flint et al., 1998; Sava et al., 2014), which implies that the number of glycinergic terminals is rather small in the immature cortex.

The analysis of the temporal profile of GABAergic synapses revealed a substantial upregulation in the average density of GABAergic synapses from approximately. 900 mm^–2^ to approximately 3500 mm^–2^ within the first postnatal week, as expected from previous publications (Blue and Parnavelas, 1983; Micheva and Beaulieu, 1996; Virtanen et al., 2018; Gour et al., 2021). Beyond confirming the delayed developmental upregulation of GABAergic synapse density in infragranular layers as compared to supragranular layers (Micheva and Beaulieu, 1996; Virtanen et al., 2018), the present study obtained for the first time a precise spatial profile of GABAergic synapses during the first 12 days of postnatal development.

The quantification of the spatial profile of GABAergic synapses demonstrated that in P0-5 slices the highest density (up to 9,800 mm^–2^) was reached in the outermost region of the brain, which represents the marginal zone/layer 1 (Ignacio et al., 1995). The dominance of GABAergic synapses in the marginal zone is in line with the previously described dominant expression of vGAT in this layer between P0-2 (Minelli et al., 2003), and with the high density of gephyrin clusters in the prefrontal cortex marginal zone at P5 (Virtanen et al., 2018). During these early developmental stages, GABAergic synapses are formed by GABAergic axons originating from the thalamic zona incerta (Dammerman et al., 2000) and GABAergic interneurons located in the marginal zone (Soda et al., 2003; Damilou et al., 2024). Furthermore, GABAergic SPn projecting toward the marginal zone (Myakhar et al., 2011) can contribute.

During this early developmental phase, the marginal zone is populated by CRn (Kirischuk et al., 2014; Causeret et al., 2021), suggesting that these neurons may represent an important postsynaptic target of early GABAergic inputs. Functional GABAergic inputs on CRn have been reported by several studies (Kilb and Luhmann, 2001; Radnikow et al., 2002; Kirmse et al., 2006; Damilou et al., 2024). In line with these observations, our analyses in ΔNp73^Cre^ reporter animals revealed an overrepresentation of GABAergic synaptic profiles close to the surface of putative CRn. On average, we observed 20 somatic and 40 dendritic synapses per CRn, which is likely even an underestimate of the total number, since only a fraction of CRn are labeled in the ΔNp73^Cre^ transgenic animals (Bielle et al., 2005; Causeret et al., 2021). In contrast, a rather low frequency of spontaneous GABAergic currents was previously recorded from CRn in slice preparations (Kilb and Luhmann, 2001; Radnikow et al., 2002; Kirmse et al., 2006). Taken together, these data either indicate a low release probability of CRn GABAergic synapses, in accordance with the high failure rate observed for GABAergic synapses in other immature systems (Doischer et al., 2008; Yang et al., 2014), or low activity levels of the GABAergic axonal projection to CRns in slice preparations. Indeed, enhancing network activity increased the rate of GABAergic PSCs in CRn up to 3.6 Hz (Kolbaev et al., 2011) and adding electrical stimulation increased the amplitude of PSCs recorded from CRn (Damilou et al., 2024) probably reflecting a high number of synaptic sites in CRn. GABAergic responses of neocortical CRn are putatively excitatory, due to a NKCC1-mediated Cl^–^ uptake (Achilles et al., 2007) and this depolarizing GABAergic signaling further serves as a pro-apoptotic signal promoting the postnatal death of CRn (Blanquie et al., 2017). Although CRn represent a major transient cell population in the neocortex of many species, including humans, very little information is available on CRn’s functional outputs. A very recent study describes CRn as transient targets of layer I interneurons before their connectivity progresses to layer 2/3 pyramidal cells, establishing sensory-driven inhibition within and across cortical columns (Damilou et al., 2024). Interestingly, aberrant survival of hippocampal CRn leads to attenuated theta oscillations in the hippocampus of adult mice (Riva et al., 2023), hinting toward a putative pacemaker function of cortical CRn during early phases of development.

The very low density of GABAergic synapses in the cortical layers below the marginal zone during the first postnatal week is in line with the very low density of gephyrin clusters at P5 described in supra- and infragranular layers of the prefrontal cortex (Virtanen et al., 2018). After P5 we then observed a considerable increase in the density of GABAergic synapses in the cortical layers 2-6, which is in agreement with the previously described developmental upregulation of GABAergic synapses in supra- and infragranular layers between P5 and P15 (Micheva and Beaulieu, 1996; Virtanen et al., 2018) and the fact that GABAergic interneurons reach their final layer position mainly at the end of the first postnatal week (Miyoshi and Fishell, 2011). Between P6 and P12 the density of GABAergic synapses in putative supragranular layers was considerably higher than in infragranular layers, supporting a delayed maturation of GABAergic synapse density in infragranular layers (Micheva and Beaulieu, 1996). In addition, the delayed development of GABAergic synapses in supra- and infragranular layers matches the observation that, apart from pioneer neurons like CRn and SPn (Kilb and Luhmann, 2001; Hanganu et al., 2002), reliable functional GABAergic synapses were observed only at the end of the first postnatal week (Daw et al., 2007; Pangratz-Fuehrer and Hestrin, 2011; Tuncdemir et al., 2016). Accordingly, GABA-dependent patterns of cortical network activity only emerge around the end of the first postnatal week (Allène et al., 2008).

Between P0 and P5 we observed a moderately elevated density of GABAergic synapses in the lowest lamina of the cortex, which probably represents the subplate at this developmental phase (Ignacio et al., 1995; Kanold and Luhmann, 2010). These GABAergic synapses likely form the basis of the excitatory GABAergic postsynaptic potentials observed in SPn already at P0-3 (Hanganu et al., 2002; Meng et al., 2014) and most probably originate from an early GABAergic network within the subplate (Qu et al., 2016; Luhmann et al., 2018).

In addition to these spatiotemporal aspects of GABAergic synapse formation, the developmental distribution along the complete somatodendritic axis of pyramidal neurons was investigated for the first time in the present study. For this analysis, we filled neurons with biocytin, which allowed a precise somatodendritic reconstruction after streptavidin-coupled staining. The morphological differentiation of the visually identified pyramidal neurons within the experimental time window of analysis is generally in line with other studies (Kroon et al., 2019). Both apical and basal dendrites showed a significant elongation during the first 12 postnatal days, while only for the basal dendrites this growth was accompanied by an increase in dendritic branching. These results suggest that the development of the basal dendrites is delayed as compared to the development of the apical ones.

In pyramidal neurons we observed a consistent increase of GABAergic synapses. At the soma the number of GABAergic synapses increases from ca. 7 to ca. 30 between P0 and P12. This trend is in agreement with a previous study from our group reporting that, before P7, few functional GABAergic synapses between putative PV interneurons and pyramidal cells were observed, whilst the coupling ratio increased until P12 (Yang et al., 2014). The number of somatic synapses observed at the end of the first postnatal week in the present study (about 17 synapses) is substantially higher than the 5 somatic synapses reported in a previous study for layer 4 neurons at comparable ages (Gour et al., 2021). We assume that this discrepancy is caused by the visual selection of neurons with a clear pyramidal morphology, and thus a putatively higher individual maturation state in the present study. In line with this assumption, at later developmental stages the number of somatic synapses (33 at P9-12 in the present study and 47 at P14 in Gour et al., 2021) was comparable between both studies. The increase of somatic GABA synapses at later developmental stages probably reflects the developmental appearance of synaptic inputs from presumptive basket cells (Daw et al., 2007; Butt et al., 2017), preferentially targeting this compartment and mediating a reliable perisomatic inhibition (Rudy et al., 2011). Structurally this increase in perisomatic inhibition is based on an emergent specification of axons toward somatic targets as a result of pruning of axon collaterals targeting dendrites (Gour et al., 2021) and functionally depends on sensory input (Lipachev et al., 2022).

In apical dendrites, the number of GABAergic synapses increased massively from approximately 50 to 400 between P0 and P12. This greatly enhanced synapse number was only partially balanced by the morphological maturation of the apical dendrite, resulting in an enhanced density of GABAergic synapses from about 0.1 μm^–1^ at P0 to 0.4 μm^–1^ at P12. Along with this strong increase in the number of GABAergic synapses, we observed a complex pattern of synaptogenesis. During postnatal development, apical dendrite synaptogenesis displayed a biphasic behavior, with a transient shift of synapses to a distal location at P6-8, followed by a condensation to more proximal positions at P9-12. We assume that this biphasic developmental trajectory may reflect the sequential formation of synapses by somatostatin-positive interneurons and the delayed formation by parvalbumin-positive basket cells (Anastasiades et al., 2016; Butt et al., 2017). The segregation of unspecific-targeting synapses to more specific soma- and dendrite-targeting synapses by antispecific axon pruning between P7 and P9 (Gour et al., 2021) most probably also contribute to this accumulation of synapses in perisomatic regions. Underlying the relatively high number of 50 dendritic synapses as early as P0-2 are likely dendrite targeting somatostatin-positive GABA interneurons (Tremblay et al., 2016), which already make synaptic contacts at early postnatal stages, as shown previously in the hippocampus (Flossmann et al., 2019).

In the basal dendrites, the number of synapses increased from approximately 15 to 300 between P0 and P12. However, this massive increase in the number of GABAergic synapses along the basal dendrites was balanced by their massive elongation during this period, thereby resulting in a stable GABAergic synapse density of approximately 0.2 μm^–1^. Furthermore, also the localization of synapses along the basal dendrite length seemed to be mostly stable between P3 and P12. These observations are in line with a previous study which also described a rather homogeneous distribution of GABAergic synapses along basal dendrites (Virtanen et al., 2018). The same study, however, also reported that the density of GABAergic synapses yet increases after P10 and particularly in the distal compartments of basal dendrites (Virtanen et al., 2018).

In contrast to the early GABAergic synapses from putative basket cells and somatostatin-positive interneurons seen during the first postnatal days, GABAergic synapses from parvalbumin-positive Chandelier neurons to the axon ignition segment of pyramidal neurons have not be found before P11/12 (Gallo et al., 2020; Gour et al., 2021). Therefore, we did not quantify the number of GABAergic synapses on distal parts of the axons. In addition, GABAergic synapses are also formed between GABAergic interneurons around the end of the first postnatal week (Yang et al., 2014), and onto NG2 cells, with a transient peak in the first two postnatal weeks (Orduz et al., 2015). The formation of these synapses probably contributes to the observed postnatal increase in GABA synapse density described in this study.

In summary, our results disclose a complex trajectory in the development of GABAergic synapses in mouse somatosensory cortex during the first 12 postnatal days. While in the first postnatal week GABAergic projections to transient pioneer neurons dominate, synapses targeting the soma and dendrites of pyramidal neurons gradually increase until P12. Substantial redistribution of GABAergic synapse along the apical dendrites probably reflects the specification of GABAergic inputs toward the adult connectivity patterns during this interval. In the adult cortex, contrasting interneuron densities with characteristic regional connectivity have been reported across different cortical regions (Wang et al., 2024; Pouchelon et al., 2021), while projection patterns, architectures and the set of interneuron types are conserved (Tasic et al., 2018). Many developmental studies (including the present study) investigated GABAergic synaptogenesis in the somatosensory cortex. However, further investigations on the developmental trajectory of GABAergic synaptogenesis are required to reveal the onset and sequence of GABAergic connectivity across different cortical region. Because GABAergic activity is an important factor for the generation of immature activity patterns (Allène et al., 2008; Egorov and Draguhn, 2013; Luhmann et al., 2014; Bollmann et al., 2023), which in turn are essential for the proper development of functional cortical circuits (Wang and Kriegstein, 2011; Kirischuk et al., 2017; Molnár et al., 2020; Warm et al., 2022), even subtle changes in the spatiotemporal trajectory of GABAergic synaptogenesis during early postnatal development can have profound impact on the function of the mature nervous system. Accordingly, identified risk genes for neurodevelopmental disorders encode for various constituents of GABAergic synaptic transmission, including GABA receptors, inhibitory synaptic proteins and chloride transporters, but also cause dysfunctions in the generation, migration and survival of inhibitory neurons (Tang et al., 2021), suggesting that malfunctions at the level of GABAergic interneurons and/or GABAergic synapses can contribute to the etiology of such diseases (Ben-Ari et al., 2012; Coghlan et al., 2012; Dienel and Lewis, 2019).

## Data Availability

The original contributions presented in this study are included in this article/Supplementary material, further inquiries can be directed to the corresponding author.
