# Supplementary material for: GABAergic integration of transient and persistent neurons in the developing mouse somatosensory cortex
Source: Front Cell Neurosci. 2025 Feb 26;19:1556174. doi: 10.3389/fncel.2025.1556174 (PMC11897519; doi:10.3389/fncel.2025.1556174)
Supplement: Supplementary file 1 [file Data_Sheet_1.docx]

Supplementary Material

|  | **P0-2** | **P3-5** | **P6-8** | **P9-12** | **Normality Tests** | **One-way ANOVA** | **Kruskal-Wallis test** |
| --- | --- | --- | --- | --- | --- | --- | --- |
| Input resistance (GΩ) | 0.9±0.13 | 0.9±0.16 | 0.8±0.13 | 0.45±0.07 | No |  | *p*=0.0023  ** |
| RMP  (mV) | -50.7±1.8 | -53.2±2.5 | -56.8±1.7 | -65.4±2.0 | Yes | *p*<0.0001  F=8.777  **** |  |
| AP threshold (mV) | -35.1±2.2 | -34.3±2.0 | -37.5±1.5 | -40.7±2.2 | No |  | *p*=0.1242  ns |
| AP amplitude (mV) | 49.9±3.5 | 52.3±3.6 | 63.4±2.3 | 66.2±3.2 | No |  | *p*=0.0002  *** |
| AP duration  (ms) | 3.6±0.36 | 3.1±0.32 | 2.5±0.15 | 1.9±0.12 | No |  | *p*<0.0001  **** |

**Supplementary Table 1:** Passive and active properties of recorded pyramidal neurons

**Supplementary Figure 1**


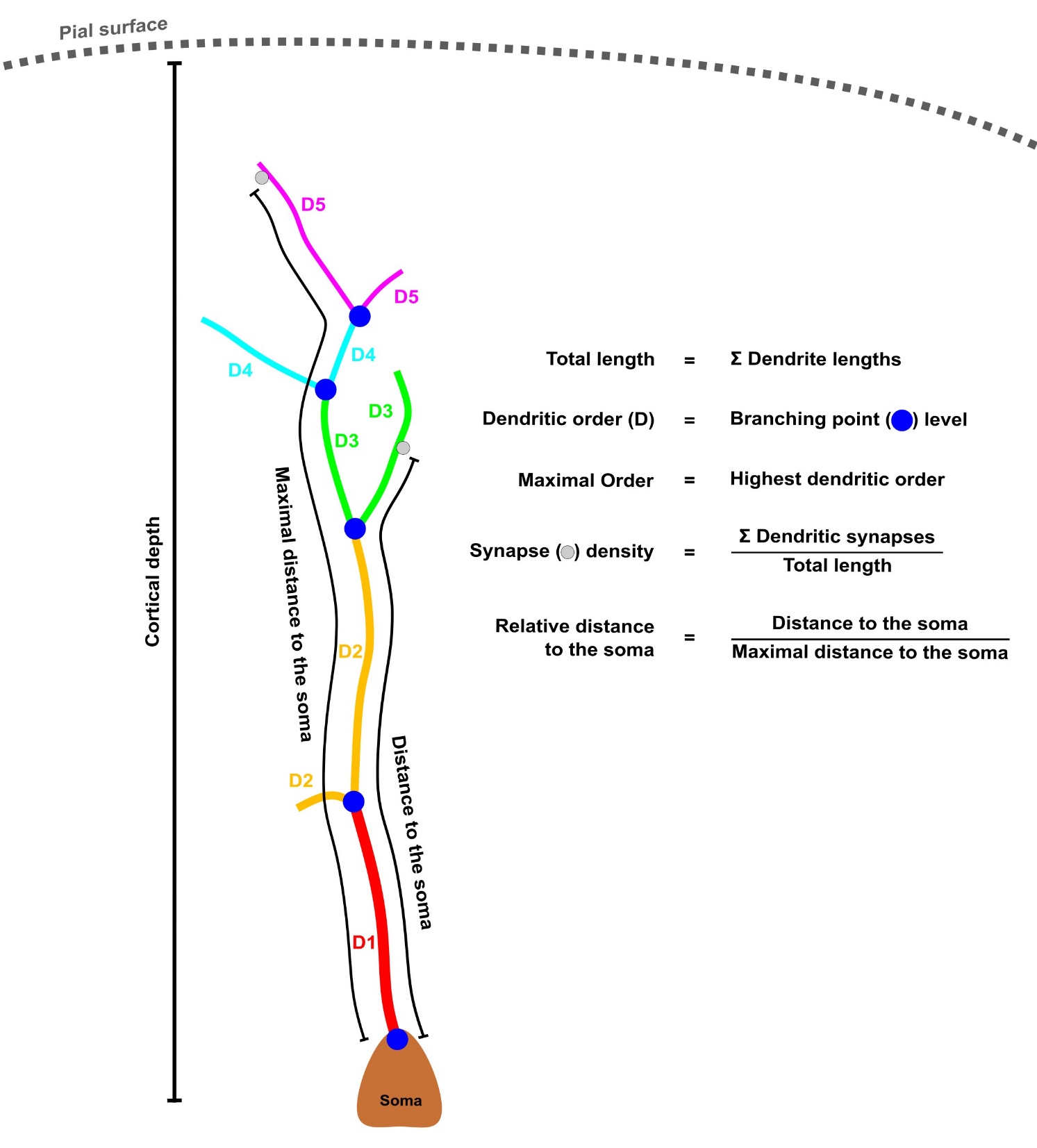


**Supplementary Fig. 1 Schematic representation of analysis of dendritic organization and GABAergic synapse localization.** After the reconstruction of single cell morphology, apical and basal dendrites were identified and analyzed recurring to Imaris software built-in functions to acquire the here depicted metrices.

**Supplementary Figure 2**


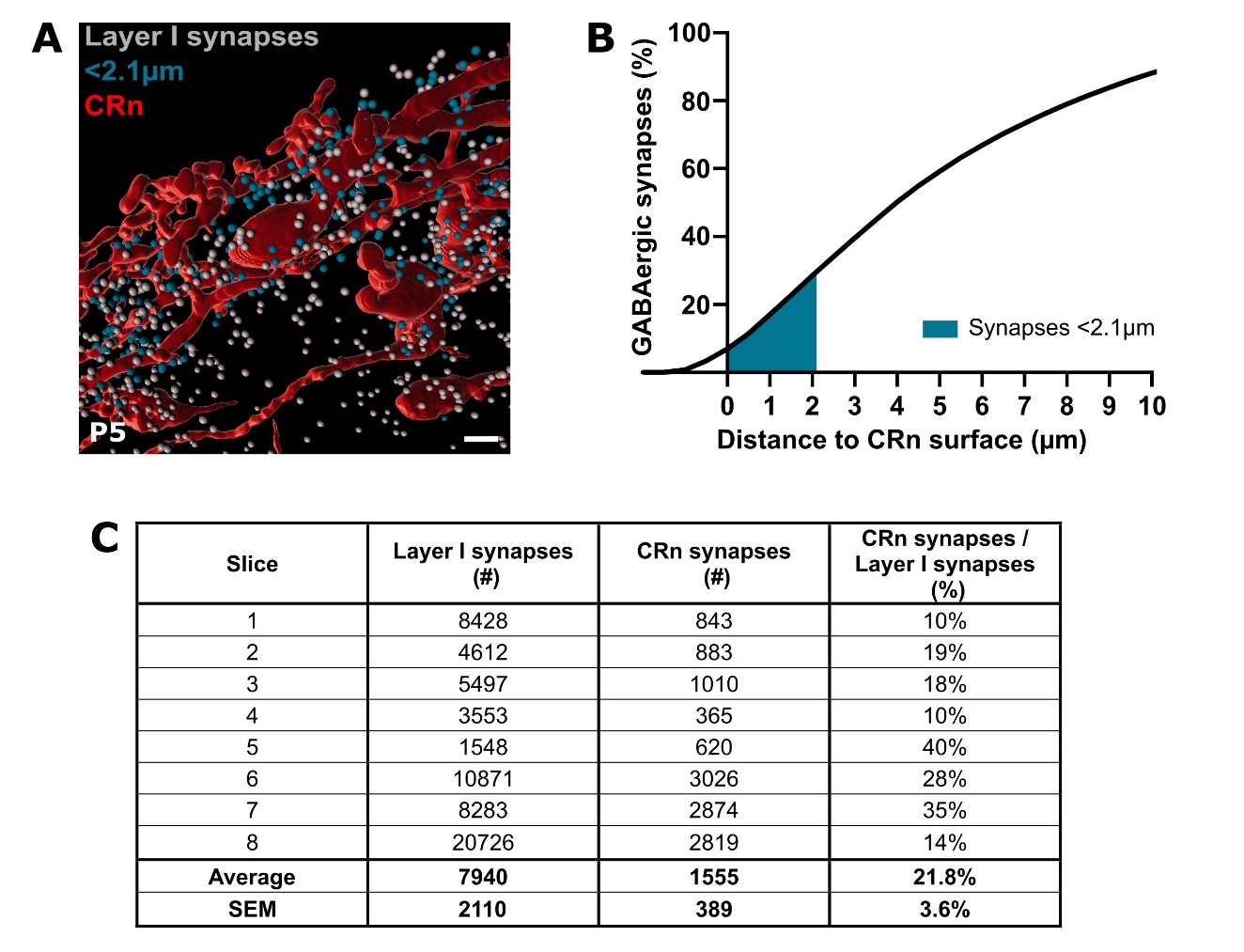


**Supplementary Fig. 2 Proximity of GABAergic synapses to Cajal-Retzius neurons (CRn). (A)** Close up of putative GABAergic synapses and CRn surface with fraction of putative GABAergic synapses as close as 2.1 µm to CRn surface colored in blue. Scale bar = 5 µm. **(B)** Cumulative percentage of GABAergic synapse plotted against the distance to CRn surface. Note, proportion of synapses at less than 2.1 µm distance colored in blue. **(C)** Tabular display of all layer I synapses, GABAergic synapses that can be reliably attributed to CRns (i.e. within 1 µm distance to reconstructed CRn surface) and the relative percentage of CRn GABAergic synapses in different slices.

**Supplementary Table 2:** Localization of GABAergic synapses on pyramidal neurons.

|  | **P0-2** | **P3-5** | **P6-8** | **P9-12** | **Normality Tests** | **One-way ANOVA** | **Kruskal-Wallis test** |
| --- | --- | --- | --- | --- | --- | --- | --- |
| Basal dendrites - Total length [μm] |  | 313±88 | 587±103 | 1109±200 | No |  | *p*=0.0144  * |
| Apical dendrites - Total length [μm] | 508±30 | 486±117 | 696±80 | 1020±130 | No |  | *p*=0.0102  * |
| Basal dendrites - Maximal order |  | 4.2±0.7 | 5.2±0.7 | 9.8±2.1 | No |  | *p*=0.0127  * |
| Apical dendrites - Maximal order | 11.3±1.7 | 9.5±1.4 | 10.8±1.1 | 9.9±0.8 | Yes | *p*=0.7384  F=0.4223  ns |  |
| Cortical depth [μm] | 822±63 | 1148±98 | 1206±69 | 1409±55 | No |  | *p*=0.0022  ** |
| Somatic synapses | 7±6 | 13±8 | 17±6 | 33±13 | No |  | *p*=0.0753  ns |
| Basal dendrites - Synapse density [μm^-1^] |  | 0.2±0.1 | 0.2±0.05 | 0.2±0.06 | Yes | *p*=0.9448  F=0.0570  ns |  |
| Apical dendrites - Synapse density [μm^-1^] | 0.1±0.03 | 0.2±0.07 | 0.3±0.04 | 0.4±0.08 | Yes | *p*=0.0275  F=3.533  * |  |

**Supplementary Figure 3**


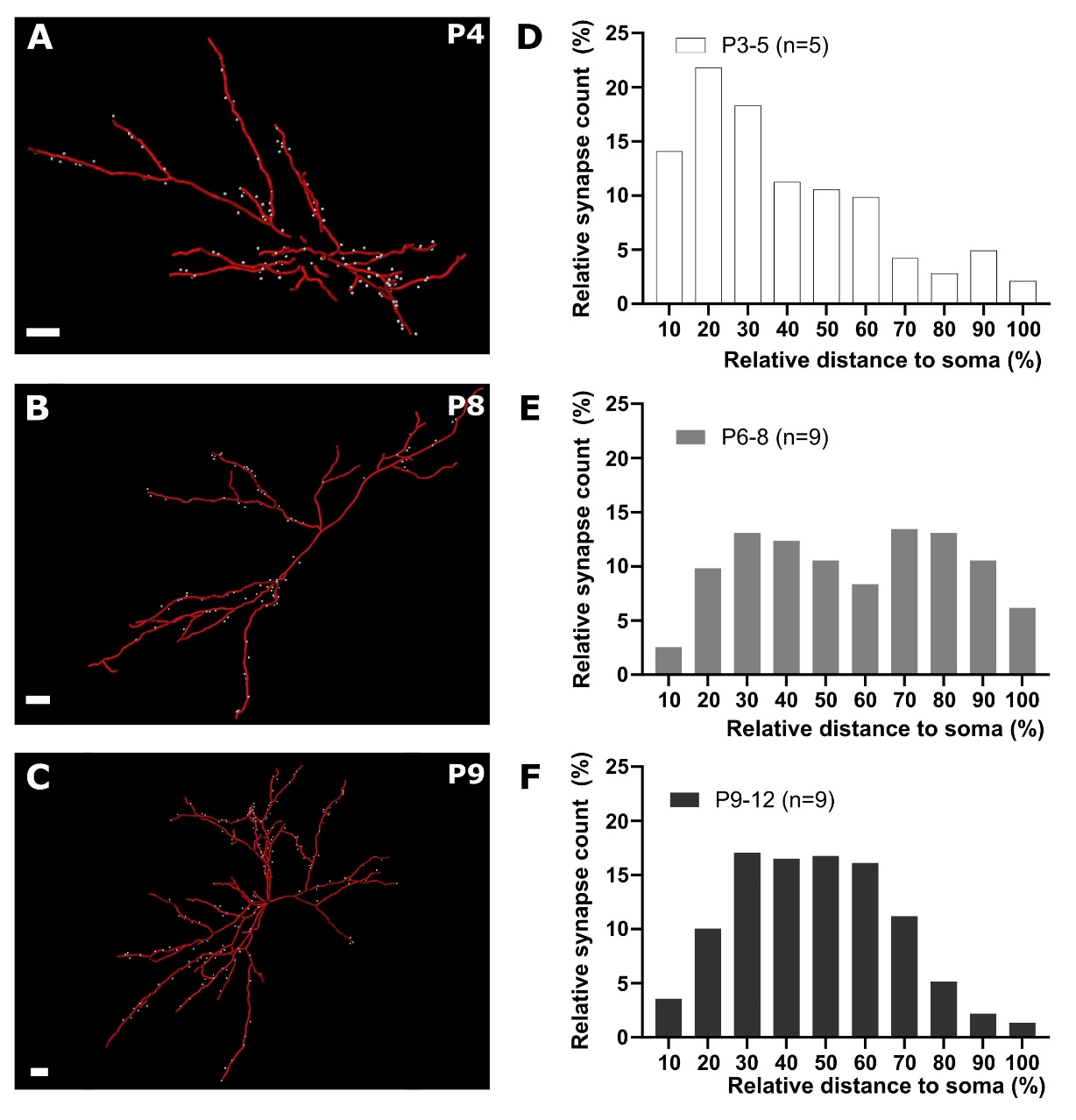


**Supplementary Fig. 3 GABAergic synapse distribution on basal dendrites during early cortical development.** **(A-C)** Representative reconstructions of GABAergic synapse positions on basal dendrites at different postnatal time points. Scale bar = 10 µm. **(D-F)** Quantification of GABAergic synapse distribution along basal dendrites between P3 and P12 plotted as relative number of synapses against relative distance to the soma. Note that at P3-5 synapses are mostly localized in close proximity to the soma. While at P6-8 and P9-12 relative prevalence of GABAergic synapses are more equally distributed between soma and distal locations.

**Supplementary Table 3:** Comparative analyses of GABAergic synapse positions on basal and apical dendrites

|  | **P3-5** | **P6-8** | **P9-12** | **Normality Tests** | **One-way ANOVA** | **Kruskal-Wallis test** |
| --- | --- | --- | --- | --- | --- | --- |
| Basal dendrites – Average distance to the soma [μm] | 29±1 | 51±1 | 70±1 | No |  | *p*<0.0001  **** |
| Apical dendrites - Average distance to the soma [μm] | 96±4 | 143±2 | 111±1 | No |  | *p*<0.0001  **** |
| Basal dendrites - Relative distance to the soma | 0.4±0.01 | 0.5±0.01 | 0.5±0.004 | No |  | *p*<0.0001  **** |
| Apical dendrites - Relative distance to the soma | 0.5±0.01 | 0.6±0.005 | 0.5±0.004 | No |  | *p*<0.0001  **** |
| Basal dendrites - Dendritic order | 3.0±0.08 | 3.9±0.06 | 7.0±0.09 | No |  | *p*<0.0001  **** |
| Apical dendrites - Dendritic order | 6.4±0.12 | 9.0±0.07 | 7.1±0.05 | No |  | *p*<0.0001  **** |
| Basal dendrites - Relative order | 0.57±0.01 | 0.59±0.006 | 0.55±0.004 | No |  | *p*=0.0003  *** |
| Apical dendrites - Relative order | 0.63±0.01 | 0.76±0.004 | 0.64±0.004 | No |  | *p*<0.0001  **** |
